# Nonexistent Objects as Truth-Makers: Against Crane’s Reductionism

**DOI:** 10.1007/s11406-016-9710-2

**Published:** 2016-04-19

**Authors:** Filippo Casati, Naoya Fujikawa

**Affiliations:** 1 University of St. Andrews, St. Andrews, UK; 2 Tokyo Metropolitan University, Tokyo, Japan

**Keywords:** Nonexistent objects, Truth-maker, Meinongianism, Possible objects, Reductionism

## Abstract

According to Meinongianism, some objects do not exist but we can legitimately refer to and quantify over them. Moreover, Meinongianism standardly regards nonexistent objects as contributing to the truth-makers of sentences about nonexistent objects. Recently, Tim Crane has proposed a weak form of Meinongianism, a reductionism, which denies any contribution of nonexistent objects to truth-making. His reductionism claims that, even though we can truly talk about nonexistent objects by using singular terms and quantifiers about them, any truth about nonexistent objects is reducible to some truths about existent objects. In this paper, we critically examine the reductionism casting some doubts on the reducibility of truths of sentences like ‘a winged pig is possible’ or ‘some winged pig does not exist’ into truths about existent objects. We also argue that the truth of such sentences can be explained by adopting a strong form of Meinongianism which admits contribution of nonexistent objects to the truth-making of such sentences.

## 1. Nonexistent Objects and Truth-Making

Meinongianism is understood as “the view that some objects do not exist, but we can generally talk about them, quantify on them, and state true things about them” (Berto 2013b, p. 115). Given this definition, Meinongianism covers any philosophical position which endorses the following two ideas:

1. Some objects do not exist.
2. We can refer to and quantify over nonexistent objects to make true statements about them.

In addition to these, contemporary Meinongians standardly hold the following third (maybe implicit) assumption:

[3] Nonexistent objects are (or, at least, are parts of) the truth-makers of sentences which contain reference to or/and quantification over nonexistent objects.

As Azzouni claims: “Contemporary Meinongians think that nonexistent objects have properties. They also think that how nonexistent objects are (what properties they have) are the truth-makers for sentences about those objects” (Azzouni 2012, p.254). For Meinongians, the so-called characterization principle (CP) guarantees the first point of the quotation, that is, the possession of properties by nonexistent objects. The version originally proposed by Meinong (1904) is called *naïve* CP and it claims that, for any characterization (any set of properties), some object which satisfies the characterization is in the domain of discourse. However, this version was vulnerable to Russell’s objections (cf. Russell 1905, pp.482–483) and it was quickly dismissed. Consequently, nowadays, there are three revised versions of CP. Some Meinongians (cf. Parsons 1980; Routley 1980) claim that, distinguishing the so-called ‘nuclear’ and ‘extra-nuclear’ properties,^1^ CP must be applied to characterizations which contain only nuclear properties. Some other Meinongians (cf. Zalta 1988) claim that there are two different ways of having properties: encoding and exemplifying. According to them, a nonexistent object encodes all the properties it is characterized as having but does not exemplify them. Finally, another group of Meinongians (cf. Priest 2005; Berto 2013a, Chap. 6.3) claims that an object has all the properties it is characterized as having not necessarily in the actual world but in some possible or impossible worlds. Even though these three versions of CP differ in details, it is certain that all of them ensure that nonexistent objects have properties (in some ways). And contemporary Meinongians like Parsons, Routley, Zalta and Priest seem to take the property-possession of nonexistent objects as contributing to truth-making of sentences about nonexistent objects. For example, even if *a* is a nonexistent object, if *a* has the property *P*, then, the sentence ‘*a* is *P*′ is true simply because of *a*’s possession of *P*. In this way, nonexistent objects can be involved in truth-making of sentences about them. Moreover, Meinongianism accepts that a nonexistent object can have properties which are not parts of its characterization. For example, some nonexistent objects instantiate such properties as *being incomplete*, *being possible*, *being thought of by someone*, and so on. Because of this, according to these contemporary Meinongians, nonexistent objects can be involved in the truth-makers of sentences such as ‘Sherlock Holmes is possible’ and so on.

Let us call a position which endorses **[1]**, **[2]** and **[3]** *strong Meinongianism*. Many contemporary Meinongians seem to endorse strong Meinongianism. However, recently Tim Crane (Crane 2013) has introduced a new form of Meinongianism (let us call it *weak Meinongianism*), which endorses **[1]** and **[2]** but *rejects* **[3]**. He proposes a *reductionist* 2 account of nonexistent objects which claims that, even though both **[1]** and **[2]** hold, truths of singular and/or quantificational sentences about nonexistent objects are reducibly explained by truths about *existent* objects. According to him, “some objects of thought exist and some do not” (Crane 2013, p.38, henceforth all page references are from Crane 2013 unless otherwise noted). Moreover, he also claims:

not only can we talk about them [nonexistent objects] by using names but we can also talk about them by using quantifiers – for instance, expressions which pick out or specify a quantity of things, such as ‘some’, ‘all’, ‘most’, ‘many’ and so on (p.16).

Sentences like ‘Pegasus is a mythical horse’ (p. 23) or ‘some biblical characters did not exist’ (p. 40) not only are true but also contain reference to and/or quantification over nonexistent objects.^3^ However, his reductionism claims that *no* nonexistent object is involved in the truth-making of such true singular/quantificational sentences about nonexistent objects. Truths about nonexistent objects are reduced to truths of existent objects. Let us see his reductionism in more detail.^4^

According to Crane, both singular and quantificational sentences receive the following standard semantic analysis, no matter whether they are about existent objects or nonexistent objects (pp. 37–42).

1. ‘a is F′ is true iff a has the property *F*.
2. ‘Some F is G’ is true iff for some object, it has the property *F* and the property *G*.

For example, ‘Pegasus is a mythical horse’ is true iff Pegasus has the property of *being a mythical horse*. ‘Some biblical character does not exist’ is true iff for some object *x*, *x* is a biblical character and does not exist, in the same way as ‘some pig is pink’ is true iff for some object *x*, *x* is a pig and pink. In this way, Crane’s reductionism admits that nonexistent objects have properties. However, reductionism claims that there is a significant limitation on what kind of properties nonexistent objects can have. They can have only *pleonastic* properties and they cannot have any *substantial* property (we will see the definitions of these two kinds of properties soon below). A property is substantial if it “constitute[s] the nature of existing things, which is open to investigation, and whose instantiation depends on more than their merely being represented” (p. 70). Introducing the notion of pleonastic property, he focuses on the idea that having a pleonastic property is always reducible to facts that are constituted by instantiation of substantial properties. The possession of a pleonastic property, *F*, by an object, *a*, depends on the truth of the sentence ‘*a* is *F*′, not vice versa. And the truth of the sentence is ultimately explained *not* by *a*’s instantiation of *F*, rather *by some existent object*’*s*/*objects*’ *having some substantial property*/*properties*, even if *a* is a nonexistent object (cf. p. 75). In this way, no nonexistent objects can be involved in truth-making, even for sentences about nonexistent objects. Pleonastic properties are not, so to speak, genuine properties: they do not have the power of truth-making. According to him, reality is constituted by only existent objects and substantial properties. Truths about both existent and nonexistent objects are explained exhaustively by reality in this sense.

Then, what properties are counted as pleonastic? According to Crane, almost all pleonastic properties are *representation-dependent*. The only exceptions are “the property of being non-existent, and logical properties like self-identity” (p. 69). Representation-dependent properties are “properties which depend upon the fact that the object is being represented in some way: in thought, language, picture, and so on” (p. 68). One of Crane’s examples is the property of *being represented as* having *P*, where *P* is a substantial property (p. 70). For example, Holmes has the property of *being represented as a detective*. Crane also regards the properties of *being fictional* and *being mythical* as examples of representation-dependent properties (p. 68).

In order to clarify the notion of pleonastic property and its relation to truth, let us discuss these examples in more details. ‘Holmes is represented as a detective’ is true. The truth-maker of this sentence consists in the actual activities of existent objects in the world, that is, the mental activities of Conan Doyle or any other reader of his novels. There is no contribution of the pleonastic property of *being represented as a detective* and the nonexistent object Sherlock Holmes to its truth. Contrary, *in virtue of the truth of the sentence*, Holmes has the pleonastic property of *being represented as a detective*. Sherlock Holmes instantiates also many other representation-dependent properties. For example, he instantiates the property of *being fictional*. However, the fact that Holmes has the property of *being fictional* is not the truth-maker of the sentence ‘Holmes is fictional’. Indeed, once again, this sentence is true because of an appropriate kind of representational activities by human beings such as Conan Doyle and the readers of his novels, which involves only existent objects and their instantiation of substantial properties. And only because of the truth of the sentence, Holmes has the pleonastic property of *being fictional*. In other words, the truth of the sentence ‘Holmes is fictional’ makes Holmes to instantiate the property of *being fictional*, not vice versa. At the beginning of section 2, we will give more explanation of pleonastic properties.

It is also worth noting that, even though Crane rejects CP, his weak Meinongianism is compatible with some versions of CP. In particular, reductionism is compatible with the following restricted version of CP. For any substantial property *P*, let us call the property of *being represented as having P* the pleonastic counterpart of *P*. Then, we can define a version of CP as saying that for any set of substantial properties, some object has all the pleonastic counterparts of the properties in the set.

So far we have confirmed what Crane’s reductionism, a version of weak Meinongianism, claims. Nevertheless, one question still remains open: is this version of weak Meinongianism better than the strong one? In what follows, we point out some defects of this reductionism. In particular, we claim that not all properties of nonexistent objects are reducible to reality in Crane’s sense (section 2.1) and that reductionism does not adequately explain the semantic behaviors of quantifiers ranging over nonexistent objects (section 2.2).

## 2. Some Problems of Reductionism

### 2.1 Is Possibility a Pleonastic Property?

The crucial claim of reductionism is whether any instantiation of a property by a nonexistent object is reducible to some instantiations of properties by existent objects. As we have seen, according to Crane, properties that nonexistent objects can have are representation-dependent properties, the property of *being nonexistent* or logical properties such as *being self-identical*. Crane claims that “[t]he representations on which these properties [representation-dependent properties] depend are part of reality, to be sure. So the task ahead of us is to explain the problematic truths about the nonexistent in terms of truth about these representations” (p. 120). This is not a trivial task, and Crane puts significant efforts to give some examples of such reduction (pp. 133–136). Concerning the property of *being nonexistent*, assuming that the singular negative existential sentence ‘*a* does not exist’ predicates the pleonastic property of *being nonexistent* to *a*, Crane claims that ‘*a* does not exist’ is true iff ‘*a* exists’ is false. And ‘*a* exists’ is made false, if it is, by the whole world (pp. 74–75). The truth-maker of a singular negative existential statement is the whole world, the way the existent objects actually are (p. 75). For self-identity, Crane claims that, no matter whether *a* is existent or not, identity statements of the form ‘*a* = *a*’ are true, because they are instances of the logical truth that for all *x*, *x* = *x* (pp. 162–167). However, these properties do not exhaust the properties that nonexistent objects can have, and this leads to a major problem for his reductionism. Let us discuss it.

Consider the property of *being possible*.^5^ Consider a winged pig. Since the properties which a winged pig instantiates are logically consistent, a winged pig is logically possible, even though it does not exist. Nevertheless, because a winged pig is possible regardless whether it is represented or not, the property of *being possible* is not representation-dependent.^6^ Of course, such a property is neither the property of *being nonexistent* nor logical properties like self-identity. Therefore, *being possible* is a property of nonexistent objects which is neither representation-dependent, nonexistence, nor a logical property. Crane’s trichotomy of properties of nonexistent objects is wrong. Crane would reply to this objection that possibility is indeed representation-dependent, by claiming as follows: nonexistent objects cannot have substantial properties like the property of *being a pig*. A winged pig is possible not because the properties it instantiates are consistent, but because it is represented as having consistent properties. However, there are two objections against the representation-dependency of possibility of objects. First of all, some possible nonexistent objects have never been thought. Consider a winged pig which has 115 tails and speaks Italian, Japanese, and English fluently. This is a possible object. Let us suppose one of the authors of this paper, for the first time in the whole of human history, thought about it on the 28th of May 2015. Although there was no representation of it, it was a possible object even before then. The modal property of the pig did not depend on representations. If so, there is no reason to reject that some nonexistent objects are now possible independently from any representations of them. Secondly, not only nonexistent but also existent objects can be possible. For example, the Empire State Building is a possible object. It is possible independently whether it is represented or not. It is possible simply because its properties are consistent (assuming that the actual world is consistent). One may claim that there are two different properties of *being possible*: one is substantial and the other is pleonastic. The Colosseum is substantially possible, but a winged pig is only pleonastically possible. We are not sure whether this move is promising or not. It seems possible to claim that this is an ad hoc supposition to maintain the view that nonexistent objects can only have pleonastic properties.^7^ And more importantly, this distinction will not dissolve the problem of reducibility.

A reductionist may accept that the property of *being possible* is a forth kind of pleonastic property so as to abandon Crane’s trichotomy. Now, given that reductionism is the universal claim that *all* true statements about nonexistent objects are reductively explained by appealing only to reality (in Crane’s sense), it is crucial for reductionism to show that true sentences which predicate the property of *being possible* to a nonexistent object are reductively explained as well. However, we are skeptical about the reducibility of the property of *being possible*. At least, it is not an easy task. Let us show this by trying to do the task for Crane.

Taking ‘a winged pig is possible’ to be equivalent with ‘some existent object is such that it is possible to be a winged pig’, a reductionist may claim that the truth of the former is reductively explained by the truth-maker of the latter. There are two different accounts for the truth of such modal statements about existent objects. For each of them, we can consider two attempts to reduce possibility of nonexistent objects to facts about existent objects and substantial properties.

The first attempt appeals to possible worlds. According to this account, ‘some existent object is such that it is possibly a winged pig’ is true (in this actual world) iff there is a possible world where an object is a winged pig and it is (a counterpart of) some existent object in the actual world (for simplicity, accessibility is ignored here). On the basis of this biconditional, one may take the possible worlds to be (at least a part of) the truth-maker of a sentence which predicates possibility to a nonexistent object. Then, what are possible worlds? Again, at least two accounts are available: Lewis’ modal realism and actualism. On the one hand, Lewisian possible worlds are existent, but it is hard to count them as a part of what Crane counts as reality. On the other hand, actualism claims that possible worlds are some composites of abstracts in this actual world. This view seems compatible with Crane’s reductionism. Given these considerations, reductionism may claim that the truth-maker of the sentence ‘a winged pig is possible’ consists of existent objects and possible worlds as abstract composites of things in the actual world. (Of course, it is well known that this actualist attempt to reduce possible worlds to the actual one is far from being unproblematic (cf. Lewis 1986, pp. 150–165). Thus, if we run together reductionism and actualism, we can, at least, say that the former faces the same problem of the latter.)

The second attempt goes as follows. First, it supposes that the modal properties of the form *being able to be such and such* are substantial properties of existent objects. Then, the truths of the statements in question (‘some existent object is such that it is possibly a winged pig’ and ‘a winged pig is possible’) are explained by the fact that some existent objects instantiate these properties. According to this attempt, the truth-maker of the sentence ‘a winged pig is possible’ consists of existent objects and (their instantiation of) certain modal properties as substantial properties.

Both attempts may work for sentences of the form ‘an/some F is possible’, given the supposed equivalence of ‘an F is possible’ with ‘some existent object is such that it is possible to be F’. However, it is not clear how other forms of sentences which predicate possibility of nonexistent objects can be reductively explained in a similar manner. For example, is a simple sentence like ‘Pegasus is possible’ reductively explained in this way? There seems to be two ways to do so. The first one is to claim that ‘Pegasus is possible’ is true iff some existent object is able to be Pegasus. But, identity is necessary and no existent object is Pegasus, therefore no existent object can be Pegasus. The second way is to claim that assuming that the name ‘Pegasus’ can be replaced by a suitable definite description, ‘the φ’, ‘Pegasus is possible’ is true iff ‘some existent object is able to be uniquely φ’ is true. The result of this proposal depends on how we specify φ. One may regard φ as *being described as such and such in the Greek myth*. An apparent problem of this option is that it fails to appropriately distinguish between possible and impossible objects. For example, let us consider the box described in the short story ‘Sylvan’s Box’ (Priest 2005, sec. 6.6). According to the story, the box is empty, but not empty as well—a small figure is in it, and not. Intuitively, the box is impossible. Now, along the line of the present option, the box is possible iff some existent object is able to be uniquely described as being empty and not empty in ‘Sylvan’s Box’. However, it may be the case that the story describes a fictional story about the actual box (as *War and Peace* is a fictional story about Napoleon). In this case, according to the present option, the box is possible. This contradicts our intuition. Of course, this problem is easily avoided by adding the consistency of the description to the right-hand side of the biconditional. However, even if this revision is made, the following problem will remain. It is quite unclear whether some existent object is actually able to be *uniquely* described as a winged horse in the Greek myth. Maybe some horses which existed in ancient Greece were able to be described as a winged horse in the Greek myth, but it is hard to see which one was uniquely so. The second option is to take φ as *being a winged horse with the properties which the Greek myth ascribes to Pegasus*. Again, we are quite unsure whether some existent object is actually able to be uniquely such a winged horse. Which object can be the unique winged horse with the properties ascribed by the Greek myth to Pegasus? Some existent horses? You? We think there is no definite answer.

Reductionism tries to avoid these problems by claiming that the truth-makers of sentences which predicate possibility to nonexistent objects contain neither nonexistent nor *existent* objects at all. In particular, a reductionist may try to explain the truths of such sentences by consistency of substantial properties. Let us label this position the *no-object approach*. For example, according to the no-object approach, ‘a winged pig is possible’ is made true by the fact that the property of *being winged* and the property of *being a pig* are consistent. Since no existent objects nor instantiation of any kind of properties are involved in truth-making of the sentence, the problems described above disappear. However, once we consider several quantificational sentences about nonexistent objects and the inferential relation among them, it turns out that the no-object approach is not available for reductionists. Moreover, such consideration reveals another problem of reductionism concerning quantifications. These problems will be discussed in the next section.

### 2.2 Are all Quantifications over Nonexistent Objects Reducible?

Consider the following statements:

(3) ᅟ

- A winged pig is possible.
- Most winged pigs are possible.
- Every winged pig is possible.

At least for someone like Crane who takes quantification over nonexistent objects in natural languages at face value, these sentences are not equivalent and thus must have different truth-makers, if they have any. Any attempt to take such quantification over nonexistent objects as genuine must be sensitive to these differences. Then, how can a reductionist distinguish the truth conditions of them, within the framework of the no-object approach, which appeals to the consistency of a set of properties?

Given the no-object approach, the following may be a suggestion. Suppose that S is the set of all sets of substantial properties which contain both the property of *being winged* and the property of *being a pig*. (3a) is true iff there is a consistent set in S. (3b) is true iff more than half of the members of S are consistent. (3c) is true iff all members of S are consistent. These truth conditions give us the following predictions (given |S| > 2): (3c) entails (3b), (3b) entails (3a), but not vice versa. This seems correct, if the quantifiers behave with respect to nonexistent objects in the same way as they do with respect to existent ones.

However, this suggestion is silent on the reason why the facts about sets of properties can be the truth-makers of such quantificational sentences about nonexistent objects. The only possible reply is that, between such sets and nonexistent objects, there is some relation responsible for this. But, then, which is this relation? An obvious answer to this question is that *sets of substantial properties are properties of* (*existent or nonexistent*) *objects*. However, once a reductionist accepts this connection between nonexistent objects and sets of substantial properties, she needs to abandon the claim that nonexistent objects can have only pleonastic properties. As we have seen, for something to be possible, it needs not to be represented as having properties. Thus, the required relation between properties and objects should not depend on representations. A substantial property is a property of an object *x* in the required sense, only if *x* representation-independently has the properties. And this leads to abandon reductionism, since if nonexistent objects can have substantial properties, they should be able to do truth-making in the same way as existent objects do truth-making, given that existent objects make true sentences about them by instantiating some substantial properties. Therefore, the reductionist should not employ the no-object approach.

Our argument so far relies on the claim that the property of *being possible* is not representation-dependent. This is plausible, given the reasons we already stated in section 2.1: for example, the Empire State Building, an actually existent object, would be possible even if no one had ever thought about it. However, a reductionist may insist on the claim that the property of *being possible* is representation-dependent and, as we suggested in section 2.1, a winged pig is possible because it is represented as having consistent properties. More specifically, to connect nonexistent objects and (sets of) substantial properties, a reductionist may propose (5) as the semantic analysis of (3), given (4):

(4) *x* is possible iff *x* is represented as being φ, where φ is consistent, for some appropriate φ.

(5) ᅟ

- For some *x*, *x* is represented as being φ, where φ is a conjunction of properties among which is the property of *being a winged pig*, and *x* is possible.
- More than half of things represented as being φ, where φ is a conjunction of properties among which is the property of *being a winged pig*, are possible.
- For every *x*, if *x* is represented as being φ, where φ is a conjunction of properties among which is the property of *being a winged pig*, then *x* is possible.

First of all, proponents of this view must give a reductionist account of the truth of the right-hand side of (4), in particular, the truth of ‘*x* is represented as φ’, where *x* is a nonexistent object. For the sake of argument, let us assume that some appropriate reductionist account for it is available. Then, (5) seems to correctly predict inferential relation among (3). This is good news for reductionists. If so, besides the problem we have stated, what’s wrong about this proposal? This reductionist account of quantificational sentences predicating possibility to objects has wrong results about their truth conditions. Consider the sentence ‘some perpetual motion machine is possible’. This sentence is false. Indeed, every perpetual motion machine is impossible, given the law of physics. Now, imagine that the first steam engine in the history is a perpetual motion machine. Of course, this imagined story is false, but it is the case that the existent engine is represented as being a perpetual motion machine in the story. Now, since the engine is an existent object, it is possible. Thus, the first steam engine, which is represented as being a perpetual motion machine, is possible. Therefore, according to the reductionist analysis, ‘some perpetual motion machine is possible’ is true. To avoid this result, a reductionist may claim that the first steam engine is impossible. (Indeed, if we assume that ‘being a perpetual motion machine’ is the appropriate φ for the first steam engine, (4) tells us that, when the steam engine is represented as being a perpetual motion machine, it is not possible.) However, this is too much even for reductionists who stick with the representation-dependency of possibility.

Reductionism also has difficulty in giving an explanation of truths of quantificational negative existential sentences like ‘some winged pig does not exist’. As we have seen, weak Meinongianism, which takes quantification over nonexistent objects at face value, claims that this sentence is true iff for some *x* (in the domain of discourse), *x* is a winged pig and it does not exist. Here a problem arises. According to reductionism, the property of *being winged* and the property of *being a pig* are substantial properties and, thus, only existent objects can instantiate them. From this, it follows that the right-hand side of the equation is never true. Therefore, according to reductionism, ‘some winged pig does not exist’ is false, and therefore ‘all winged pigs exist’ is true. But the former is true and the latter is false.

A reductionist might reply by claiming that we do not need to specify the truth-makers of quantificational (positive/negative) existential statements in this way. There can be other ways to specify their truth-makers which predict the correct truth conditions. Indeed, the reductionist could claim that the truth-maker of ‘some winged pig does not exist’ is the whole world, in particular, the fact that no existent object in the world instantiates both the substantial properties of *being a pig* and *having wings* at the same time. Any other forms of negative existential states may be understood in this way: they are made true by the fact that no existent object in the world is such and such. However, this is incorrect. In particular, this proposal leaves inferential behaviors of such sentences unexplained. For example, if we take the quantification over nonexistent objects seriously ‘some winged pig does not exist’ is not equivalent with ‘three winged pigs do not exist’. But, if the proposal is correct, they have the same truth-maker, that is, the whole world, and, thus, are equivalent. Moreover, if the reductionist appeals to sets of substantial properties to capture the semantic relations between them, the same problem mentioned before arises.

A reductionist may hold that quantificational negative existential statements are representational dependent by claiming that, for example, ‘every winged pig does not exist’ is true iff everything which is represented as being a winged pig (and being such and such) does not exist. However, this again gives a wrong prediction. We can imagine a fictional story about an existent actual pig according to which it is a winged pig. Given such a fictional story, the alleged representation-dependent analysis of negative existential sentences predicts that ‘every winged pig does not exist’ is false.

Not surprisingly, strong Meinongianism does not suffer these problems. This is because it admits that nonexistent objects can contribute to truth-making. For instance, consider strong Meinongians *à la* Parsons. Since the property of *being winged* and the property of *being a pig* are nuclear properties, his version of CP ensures that the domain of discourse contains a bunch of winged pigs. Further more, let us define *x*’s being possible by the consistency of properties which *x* instantiates. Then, the truth of (3a) ‘a winged pig is possible’ could be simply explained by appealing to the fact that some nonexistent object has the property of *being a pig*, *being winged* and the property of *being possible*. Secondly, according to this position, (3b) ‘most winged pigs are possible’ is true iff more than half of the members of the set of all winged pigs are possible. Finally, the sentence (3c) ‘every winged pig is possible’ is true iff all the members of the set of all winged pigs are possible. And these truth conditions correctly predict the entailment relations among (3a), (3b) and (3c), given that the set of all winged pigs contains more than two pigs. The truth conditions of quantificational negative existential statements are explained in the similar manner.

## 3. Conclusion

The present paper has shown the following: first, it is difficult to reductively explain the instantiation of the property of *being possible* by nonexistent objects. Secondly, there is a serious tension between admitting quantification over nonexistent objects, on the one hand, and the reductionist approach which tries to eliminate nonexistent objects from truth-making, on the other hand. If the reductionism is abandoned, and only the reductionism is weak Meinongianism, there seems to be only two options. The first one is to adopt strong Meinongianism by admitting truth-making by nonexistent objects. The second option is to abandon Meinongianism altogether by rejecting genuine reference to and quantification over nonexistent objects. In short, if reductionism is the only option for weak Meinongianism, then to be a strong Meinongian, or not to be a Meinongian at all - this is the question.^8^

## Footnotes

1 For more details about the distinction between nuclear and extra-nuclear properties, see Parsons (1980, pp.17–22; pp.166–174).

2 The anonymous referee has suggested that this terminology is not explanatory enough because it leaves open the problem about what exactly is being reduced. Thus, the referee has suggested another terminology, namely *ersatz* noneism. Even though we agree on the possibility of having more explanatory way of labeling Crane’s position, we prefer to stick on the one used by Crane himself.

3 Crane insists on using the term ‘reference’ for the relationship between a name and an *existent* object. If the object does not exist, the name is simply ‘about’ its object, and does not refer to it (cf. p.9). In this paper we use the term ‘reference’ as follows. When one talks about an object by using a singular term, then the expression refers to the object. In our terminology, Crane admits reference to nonexistent objects.

4 Someone might worry that, even though our arguments presuppose a conception of truth-maker, some philosophers reject this conception. Of course, discussing about the validity of truth-making is an important issue but it does not concern the primary aim of this paper. It is important to underline that, as in the case of Crane’s reductionism (cf. p. 119), our usage of truth-making is not committed to any specific account of truth-making but is a naive and general notion of it.

5 We interpret the property of *being possible* as the property of *being classically possible*. An object is classically possible iff it does not violate classical logic, in particular, if the set of all properties of the object is consistent.

6 The anonymous referee has suggested that, if someone takes possibility as conceivability (let us call this conceivability-possibility thesis), this claim might be contentious. First of all, there are influential debates about this conceivability-possibility thesis. For some arguments against this thesis, see Hill and Mclaughlin (1999) and Soames (2005, p.196-209). Secondly, even if possibility is wrapped up with conceivability, it does not necessarily follow that the property of *being possible* is representation-dependent: it depends on how representation-dependency is understood. According to one understanding (*à la* Crane), the property of *being possible* is representation-dependent iff any object has it only relative to some actual representation which is given by some cognitive agent. However, according to conceivability-possibility thesis, *x*’s being *F* is possible if it is not eliminated by a priori consideration for *x* to be F and the antecedent of this conditional does not entail that *x* is actually represented by someone. Proponents of conceivability-possibility thesis can accept that something is possible without being represented by anyone.

7 Crane’s pleonastic properties significantly overlap with Meinogians’ ‘extranuclear’ properties (see footnote 1). *Being fictional*, *being mythical*, *being nonexistent* and *being represented by someone* are pleonastic for Crane and all of them are categorized as extranuclear properties in Parsons (1980). If *being possible* is pleonastic for Crane, then this is also a positive exemplar of the overlap in question. (The property of *being existent* might be an exception: it is extranuclear for Meinongians, but Crane might take it as substantial). If Crane’s distinction between substantial and pleonastic properties is not ad hoc and has its own justification, some Meinongians may divert this distinction to justify their distinction between nuclear and extranuclear properties. Therefore, if, as Crane claims, the distinction between nuclear and extranuclear properties is ad hoc and not justifiable, then the distinction between substantial and pleonastic properties will be ad hoc as well.

8 We would like to thank for their helpful comments Toru Genka,Takuya Niikawa, Yasushi Ogusa, Graham Priest, Masahiro Takatori, Genki Uemura, Zach Weber and the anonymous referee.
